# Experiencing Traumatic Violence: An Interpretative Phenomenological Analysis of One Man’s Lived Experience of a Violent Attack Involving a Knife

**DOI:** 10.3390/bs15010089

**Published:** 2025-01-20

**Authors:** Zoe Partington, R. Stephen Walsh, Danielle Labhardt

**Affiliations:** Psychology Department, Manchester Metropolitan University, Manchester M15 6GX, UK; r.walsh@mmu.ac.uk (R.S.W.); d.labhardt@mmu.ac.uk (D.L.)

**Keywords:** traumatic violence, interpretative phenomenological analysis, post-traumatic growth, victim, knife-enabled violence, social identity model of identity change, public health approach

## Abstract

A review of the violent knife crime literature suggests that the experiential perspective is one which has not been addressed in academic study. The research presented hereafter aims to address this literary gap and generate transferable knowledge relevant to the lived experience of violent knife crime. The experiential study of the single case within psychological research involves detailed examination of a particular event. Participant ‘J’ is the survivor of an extremely violent attack, involving the use of a knife, in his own home. J’s experience was analysed using Interpretative Phenomenological Analysis with reference to elements of the lifeworld: temporality, spatiality, intersubjectivity, and embodiment. Three themes were identified: 1. switching from past to present tense when relaying traumatic experience; 2. The presence of redemption sequences; and 3. making sense as a temporal process, which included an additional two subthemes—‘The long journey’ and ‘Seeking belongingness’. This case emphasises that the traumatic event is conceptualised as one part of a longer journey towards recovery, and that recovery itself is central to the experience of violent knife crime. Finally, the need to understand recovery as temporal process highlights the need to provide victims with appropriate support in order to avoid negative outcomes.

## 1. Introduction

Violent knife crime (VKC) has been evident in news reporting and the academic literature since the early 2000s (Williams & Squires, 2022). Despite consistent media attention and the persistence of VKC in the UK, there is currently no Home Office definition for knife crime or indeed of VKC (Williams & Squires, 2022). Therefore, for the purpose of the current research we have devised a working definition for VKC as: the intentional and actual use of physical force or power using a knife or sharp instrument against another person or persons that results in injury or death.

Research on VKC has predominantly focused upon risk factors for becoming involved as a victim or perpetrator (Browne et al., 2022; Cook & Walklate, 2020; Haylock et al., 2020; Wood, 2010) and the motivations and justifications for knife carrying (Brenner, 2022; Gilbert & Sinclair, 2019; Harding, 2020; Phillips et al., 2022; Squires, 2009; Stephen, 2009). Risk factors include being male (Ajayi et al., 2021; Bailey et al., 2020; Lemos, 2004; Vinnakota et al., 2022), deprivation and poverty (Haylock et al., 2020; Phillips et al., 2022; Reilly et al., 2023), exposure to gangs (Harding, 2012), adverse childhood experiences, and mental ill health (Haylock et al., 2020). Motivations for knife carrying include a fear of crime and a need for protection (Foster, 2013; Harding, 2012; Lemos, 2004; Ramshaw & Dawson, 2022), feelings of mistrust towards police and other agencies (Foster, 2013; McVie, 2010; Traynor, 2016), and peer influence and status (Lemos, 2004). Further, the majority of the literature on knife crime in the UK is focused on adolescents and young adults (Brenner, 2022; Clement, 2010; Skarlatidou et al., 2021; Wood, 2010). It is therefore argued that risk factors and knife-carrying motivations for young people are well established within the existing literature and that research should be conducted that goes beyond these themes.

In contrast to the existing literature, the present study presents an alternative approach to the study of knife crime to those commonly explored. Currently, the academic literature is lacking the insight gained from the detailed analysis of the lived experience of those who are victims of VKC. A database search was conducted on 4 April 2024 (ProQuest, Science Direct, SAGE Journals) using the search terms “‘lived experience’ OR experiential OR involvement OR ‘first-hand’” AND “‘knife crime’ OR ‘knife-enabled’ OR ‘sharp instrument’ OR ‘knife violence’” AND “’United Kingdom’ OR UK OR England or Ireland OR Scotland OR Wales’” to identify research specifically addressing VKC in the UK. Searches were limited to UK papers because the focus of the study is to better understand the nature of VKC in the UK; specifically, to understand how to prevent it. Two pieces of academic research were identified; however, these did not explore the lived experience of actual events of VKC. Instead, the identified literature investigated the lived experience and perceptions of VKC from the perspective of young people in general and the police (Skarlatidou et al., 2021), and the lived experience of stop and search practices (Deuchar et al., 2019). It is therefore our argument that the detailed examination of the lived experience of an event of VKC is lacking from the VKC academic literature.

According to Cook and Walklate (2019), victims’ stories offer valuable insight into the experience of victimisation and harm, injustice, resilience, and recovery. Studying the lived experience of violent crime can facilitate a more in-depth understanding of the individual impact of VKC victimology and thus generate transferable knowledge. To this end, we present herein the experiential analysis of a single case of VKC victimisation.

The importance of the contribution of lived experience to the academic literature on VKC victimisation can best be understood if some consideration is given to the elements of a public health approach to tackling VKC. Public health approaches are arguably the most effective at addressing knife crime and take a preventative stance to addressing social issues (Sethi, 2010). Such an approach is based upon research which informs the implementation of primary, secondary, and tertiary prevention strategies (Bullock et al., 2023; Neville et al., 2015). Primary prevention interventions focus on the prevention of violence prior to the emergence of risk factors, secondary prevention interventions address the issue once risk factors have emerged, and tertiary prevention is aimed at understanding violence after its occurrence to prevent its reoccurrence in the future (Haegerich & Dahlberg, 2011; Neville et al., 2015). The purpose of the current research is to inform future research directions that may, in turn, inform tertiary prevention strategies, via the analysis of a single case of VKC. Post-traumatic growth (PTG) and post-traumatic stress (PTS) are also considered. Research indicates the presence of victim–offender overlaps within the context of knife crime, which suggest that individuals who engage in crime are more likely to become victims and vice versa due to shared risk factors (Bailey et al., 2020). Additionally, the knife crime literature indicates that previous victimisation is a risk factor for engagement in knife crime (Haylock et al., 2020).

Analysis of the lived experience of VKC victimisation may highlight specific elements of experience relevant to victims that make them vulnerable to future offending or re-victimisation. The manner in which victims internalise victimhood as a social identity process may be relevant to this process, allowing insight into the mechanisms that encourage the internalisation of victim social identity. Research indicates that identification with victim identity, as opposed to survivor identity, has a greater negative impact on the emotional state of males who have experienced sexual assault in comparison to their female counterparts (Boyle & Rogers, 2020). In the domain of health, research suggests that the appraisal of symptoms and a salient social identity has a profound impact on wellbeing outcomes (S. A. Haslam et al., 2009). According to academic research, individuals who are encouraged to self-categorise as elderly are more likely to believe that they experience symptoms of hearing loss, which is associated with being older (St Claire & He, 2009). Additionally, individuals with asthma are more likely to access medication if they identify strongly as an asthma sufferer (Adams et al., 1997). Therefore, the act of self-categorisation may also have a profound impact on the wellbeing of victims of VKC, those who identify with victim social identity may experience more negative trauma outcomes because they may be more likely to accept the symptoms of PTS associated with victims. Alternatively, victim social identity may actually be useful, as victims may be more open to accessing the services they require to facilitate recovery. Analysis therefore may highlight the tendency towards or avoidance of such identification and its impact on wellbeing, thus illustrating ways that social identity processes could be relevant to the treatment of victims. Additionally, analysis may highlight the protective factors and elements of experience that promote PTG preventing individuals from future involvement, and thus enable the conceptualisation of further research to inform preventative strategies that enable positive outcomes.

### 1.1. Post-Traumatic Growth

PTG refers to the presence of positive psychological outcomes and changes following traumatic experiences (Jayawickreme et al., 2021). Individuals who experience PTG may continue to be emotionally impacted by their experience but domains such as personal strength, sense of self, future goals, connection to others, and behaviours are subject to positive reconfiguration (Joseph, 2013; Kinsella et al., 2015). Such positive outcomes occur because of the struggle, which manifests as PTS, that is endured following experiences of trauma or challenging life circumstances (Tedeschi, 2018). The presence of PTG does not necessarily indicate the absence of symptoms of PTS; instead, PTG and PTS often go ‘hand in hand’ (Joseph, 2013), with the emergence of PTG believed to occur due to efforts to adapt to PTS (Muldoon et al., 2019a). Additionally, moderate levels of PTS are suggested to be necessary for the level of cognitive processing required for PTG to occur (Joseph, 2013).

The current study analyses a single case study using Interpretive Phenomenological Analysis (IPA). While some may question the use of single-case studies within scientific research, their value lies within the depth and richness of the data they produce. One of the foundational philosophies within IPA, phenomenology, has previously influenced the experiential study of neuropsychology through single-case study (Hull, 2013); in particular, within the works of Alexander Luria and Oliver Sacks. Luria recognised the importance of experiential elements of neuropsychological pathologies, studied through the analysis of the single case (Proctor, 2022), thus highlighting the scientific potential of ideographic study. He strove to combine the objective and the scientific with the subjective, romantic aspects of reality to propose a unified approach to neuroscientific study, which he termed “Romantic Science” (Sacks, 2014). Central to such an analysis is the detailed examination of the narrative account. According to Sacks (2014), analysis of human experience “demands a narrative structure and sensibility of science”. (p. 527). Inspired by Luria’s works, Sacks utilised case studies to integrate the scientific and human elements of neuropsychological study (Hull, 2013) and in doing so demonstrated the potential for the detailed examination of the single case to highlight psychological concepts. The works of Luria and Sacks demonstrate the richness of data that can be obtained from the qualitative study of the individual case study with emphasis on the lived experience of the individual. Such an approach aligns with the idiographic principles of IPA and thus it was decided that the detailed examination of the single case would be appropriate for the investigation of the lived experience of VKC. While single-case-study research may be critiqued based on a lack of generalizability, the intention of IPA is not to produce generalizable findings. Instead, IPA is concerned with the production of transferable findings. The work of Luria and Sacks demonstrates its potential to contribute to understanding, and in the case of the current research, inform directions for future research. Indeed, Smith et al. (2009) state that “A good case study, with an insightful analysis of data from a sensitively conducted interview, on a topic which is of considerable importance to the participant, is making a significant contribution to psychology.” (p. 38). To access the experiential elements of a VKC experience using a single case, the research presented here utilises the elements of the lifeworld to understand one man’s narrative account of the experience of VKC victimisation.

### 1.2. Theoretical Concepts

#### 1.2.1. The Lifeworld

Analysis of lived experience can be conducted by giving equal attention to the analysis of elements of the lifeworld within narrative accounts. The lifeworld refers to the world which is experienced by humans and through them (Hemingway, 2011). The lifeworld is relational, meaningful, and experienced (Todres et al., 2007). It is the world in which individual experience is situated and from which meaning can be obtained (Ashworth, 2016). VKC is traumatic for victims, perpetrators, and witnesses. Trauma directly impacts an individual’s sense of self and the ways in which they relate to and interact with the world (C. Haslam et al., 2018). Therefore, the ways in which individuals make sense of their experience of trauma and the resulting psychological impact can be interpreted via aspects of the lifeworld within their accounts. Experience in the lifeworld can be understood and explored through temporality, spatiality, intersubjectivity, and embodiment (Hemingway, 2011).

**Temporality.** Temporality refers to an individual’s experience and perception of time (Hemingway, 2011). An hour can pass in the blink of an eye while a moment can seem to last an eternity. This element of the lifeworld relates to the temporal flow of experience (Ashworth, 2016). Thus, an experiential study should consider how participants perceive time, duration, and biography, and how this impacts their sense-making and experience (Ashworth, 2016).

**Spatiality.** Spatiality refers to proximal existence in relation to other objects, entities, and individuals. It considers closeness, space, and positioning within the lifeworld and what this means to the individual (Todres et al., 2007). Spatiality can be understood as going beyond the physical, extending to geographical perceptions and the social norms and meaning associated with places (Ashworth, 2016).

**Intersubjectivity.** This is concerned with the relevance of, and relationship with other people. It includes their involvement and significance within experience, and how others are affected by situations (Ashworth, 2016). Intersubjectivity refers to the concept that the lifeworld is relational, one which is shared with other people, and refers to the meaning that is attached to their presence or the things they leave behind, such as ideas, objects, and memories (Vargas, 2020). Analysis of the meaning derived from interaction with others is relevant here as it indicates the impact of social experience; as is the influence of culture and tradition on the view of the self (Hemingway, 2011).

**Embodiment.** This refers to the body and its experience within the lived world (Todres et al., 2007). Consciousness is an embodied phenomenon and meaning is derived from the interaction between the body, the environment, and others in it (Hemingway, 2011). Physical sensation and sensory input are integral to embodiment, as are concepts such as gender, disability, and emotion (Ashworth, 2016).

#### 1.2.2. Social Identity Approach

The social identity approach (SIA) is a social psychology metatheory, useful in the explanation of a wide range of social phenomena as products of group processes (Abrams, 2015). SIA is inclusive of Social Identity Theory (SIT) and Self-Categorisation Theory (SCT). SIT is concerned with the meaning and self-esteem derived from group membership (Tajfel & Turner, 1979), while SCT relates to the ways in which individuals define themselves in terms of individual and group identification (Turner, 1985). The Social Identity Model of Identity Change (SIMIC) posits that life changes, such as traumatic experiences, represent identity transitions (Praharso et al., 2017) and an individual’s ability to cope with the change is dependent on the individual’s readiness to identify with old and new groups (Jetten et al., 2009).

Change that results in loss is experienced as negative, change that results in gain is experienced as positive. The SIMIC proposes that significant life changes involve the loss of old social identities and stress that occurs due to social identity loss can be mitigated if new social identities are embraced, thus reducing the quanta of identity loss (Jetten et al., 2009). Research indicates that the ability to maintain multiple social identities (social identity continuity) following a stressful experience protects wellbeing (Praharso et al., 2017). Additionally, life transitions are perceived as threatening to wellbeing when identity loss is expected, and identity loss mediates the relationship between the source of stress and reduced wellbeing (Praharso et al., 2017). Relevant to the study of traumatic experiences, trauma pathways and outcomes entail elements of social identity discontinuity and social identity loss, which in turn impact accessibility of social identity resources such as support, solidarity, and control (C. Haslam et al., 2021).

Research suggests that the occurrence of PTG may be best understood as being social identity-based (Muldoon et al., 2019b) and that social identity change may promote meaning-making and facilitate PTG (Kinsella et al., 2015). Furthermore, and consistent with SIMIC, the development of new social identities and social group identifications (social identity gain) are suggested to be related to differences in trauma outcomes with regard to the severity of PTS symptoms and the presence of PTG (Muldoon et al., 2019a). In addition, shared social identity and establishing a shared common fate with others is considered to be related to positive trauma outcomes (Drury & Williams, 2012). Thus, in the context of the current study, an analysis of social identity continuity/discontinuity and social identity gain/loss following traumatic experience offers useful conceptual tools to contemplate the presence or absence of wellbeing and may be indicative of PTS and/or PTG.

#### 1.2.3. Redemption Narratives

Narrative identity refers to the way in which individuals make sense of and give meaning to their lives via the internalisation of evolving and self-defining stories (McAdams et al., 2006). Narratives situate a life ‘in time’ (McAdams & McLean, 2013) and also within the lifeworld as they include plots, themes, characters (intersubjectivity), scenes, and settings (spatiality/embodiment). The narrative self, or personal myth, is a continually developing inner story revealed to the self and to others throughout the lifespan (McAdams, 1993). The study of narrative identity, via the narrative accounts of individual experience, enables the identification of features and dimensions of experience which, in turn, give insight into the personal myths and narratives of individuals (McAdams & McLean, 2013). As such, the way in which individuals make sense of their stories alludes to the way in which they make sense of themselves and their experiences.

Redemption sequences within narrative accounts are those in which the narrator describes circumstances as evolving from negative to positive (McAdams, 2006; McAdams & McLean, 2013). Research on addiction recovery has noted the presence of redemption narratives in which individuals narrate social identity loss with peers and renewal via social identity gain with support groups (Dingle et al., 2015). While the concept of redemption may seem at odds with the study of victimisation, because it suggests a need to be redeemed from some wrongdoing, this is not the intended assertion within the presented analysis. Previous research has highlighted that redemptive sequences within narratives enable victims to make sense of a painful and traumatic experience and give meaning to their future, thus enabling victims to move on positively (Cook & Walklate, 2019). Thus, the current study highlights the presence of redemptive sequences within the narrative account of a victim of VKC and how this may indicate the presence of, or potential for, PTG.

### 1.3. The Present Study

The present research applies a consideration of the lifeworld, the SIA, and redemption sequences to an analysis of one man’s experiential account of VKC. The aim is to produce transferable knowledge that can inform public health prevention strategies by understanding the psychological processes relevant to victims. Ultimately, the aim of the research is to answer the question ‘What is the lived experience of VKC?’

## 2. Materials and Methods

### 2.1. Procedure

Ethical approval was granted by the institution (IRB number: 42440). J was provided with an information sheet prior to the interview and completed a consent form. Regarding the interview length, Alase (2017) notes that interview length within IPA research should be between 60 and 90 min. The interview was conducted remotely using Microsoft Teams and lasted approximately one hour and twenty minutes. Charlick et al. (2017) provide an example of IPA research conducted based the upon a single interview of similar length (1 h and 30 min) demonstrating that detailed analysis is possible with data derived from an interview of similar duration. J was given the opportunity to ask questions about the research both prior to and following the interview. The interview was recorded and transcribed using Microsoft Teams and the transcription was edited to ensure accuracy by the interviewer in the days following the interview.

### 2.2. Participant

Single-case-study research involves the meticulous and extensive examination of a particular experienced event (Schoch, 2019). It is suggested that particularly powerful data can be obtained via single case studies and that data quality, and not quantity, is the important factor in the study of human experience (Smith et al., 2009). The current study utilised the single-case approach in the context of one man’s experience of being a victim of a premeditated, prolonged, and violent attack involving the use of a knife, amongst other attack methods. The use of the single case aligns with the idiographic principals of IPA, which emphasises the subjective lived experienced as opposed to generalisable findings derived from large samples (Smith et al., 2009). IPA’s focus is on the detailed exploration of individual experience, which enables a nuanced, in-depth understanding of the individual’s sense-making of a particular experience (Smith et al., 2009). In utilising the single case in the context of VKC, personal meaning-making is explored through elements of the lifeworld and interpreted through the application of psychological theory.

Participant ‘J’ was recruited after he was directed to the researcher’s recruitment material through existing professional networks. The nature of J’s lived experience is such that the preservation of his anonymity is of the utmost importance. Therefore, any identifying information is omitted from this report with only general participant details being included. J introduced himself to the interviewer (ZP) as a 55-year-old man, who grew up with both of his parents and four siblings. J described the area he grew up in as ‘a really nice area’ and while he described his early life with fondness, he noted that a challenge for him during childhood was the realisation at the age of 9 or 10 that he is gay.

Later in his life, J was subjected to an unprovoked, prolonged, and violent attack in his own home. The offender was an individual he had met using online dating apps and whom J had met on other occasions prior to the incident. After a small number of encounters, the two naturally drifted apart and contact had ceased until the day of the VKC.

### 2.3. Analysis

#### Interpretative Phenomenological Analysis

IPA is a qualitative method of research which concerns itself with the lived experience of participants to produce transferable knowledge (Smith et al., 2009). Smith et al. (2009) assert that there is no ‘right’ way to conduct IPA, and that it is a recursive and iterative process. Central to IPA is the concept of the double hermeneutic, presented hereafter through the researcher’s sense-making of J’s own sense-making throughout his account. In integrating the results and discussion, the double hermeneutic is made transparent and accentuates the narrative that J’s recovery from traumatic violence was a temporal process, and one which represents a process of social identity change.

IPA was conducted, using a single-case-study research paradigm, in line with the guidance from Smith et al. (2009) who detail four phases necessary to conduct effective IPA: 1. reading and rereading while highlighting significant statements; 2. exploratory noting; 3. The construction of experiential statements; and 4. searching for connections and themes across those experiential statements. In stage 1 of the process, the transcript of J’s interview was read in its entirety several times to facilitate immersion in the data, while highlighting areas of significance. The purpose of this stage was immersion into J’s world without formulating conclusions, while documenting initial responses to the data (Gonder & Clarkson, 2024). Stage 2 involved returning to the transcript and making exploratory notes, which included descriptive, linguistic, and conceptional notetaking. To allow for analysis which was independent from the narrative flow of J’s account, the transcript was scrutinised from start to finish, line by line, paragraph by paragraph, and backwards (Smith et al., 2009). Within this stage, careful consideration was given to ensuring the connection between J’s words and the researchers’ interpretation of J’s words was not compromised (Eatough et al., 2008). Stage 3 involved a shift from the interview transcript to the analysis of exploratory notes to construct concise experiential statements. Experiential statements were collected in a table, in the order they arose in the interview, to enable the next phase of analysis (Smith et al., 2009). Within phase 4, each experiential statement was considered with equal importance, and connections made to group experiential statements into clusters of personal experiential themes (PETs) before assigning each of the PETs a title (Smith et al., 2009). To ‘achieve excellence’ with regard to IPA, the analysis was conducted to ensure the inclusion of four qualities deemed hallmarks for high-quality IPA; the construction of a compelling and unfolding narrative; the production of an experiential or existential account; detailed analytical consideration of participant’s words; and attending to divergence and convergence (Nizza et al., 2021). Initial coding was conducted by the primary researcher. Preliminary themes were identified, then reviewed by the second author, and revised until it was agreed that the themes identified were relevant and valid.

Positionality refers to the stance of researchers with regard to the social, political, and cultural context of the research topic (Savin-Baden & Howell, 2012). The acknowledgement of researcher positionality is crucial in qualitative research because preconceptions based on assumptions, personal values, and experience may impact the research process. The researchers involved in the current study had no previous direct experience of VKC. However, they recognised that exposure to media portrayals of VKC may influence analysis. Analysis was conducted by the primary researcher (ZP) and throughout this process a reflective journal was utilised to document and limit the influence of VKC preconceptions.

The process of bracketing off preconceived notions within IPA is complex, due to the interpretative nature of IPA. Instead, engagement in reflexivity via reflective journaling enabled the primary researcher to evaluate how positionality may influence the interpretative analysis of J’s experience. In addition, while member-checking is not explicitly included in the process of IPA, as detailed by Smith et al. (2009), J accepted the opportunity to review the first draft of the research report and agreed that the findings were an accurate interpretation of his experience.

With regard to the current research, it was hypothesised that aspects of the lifeworld would be evident in Participant J’s narrative account. Through the application of lifeworld existentials (temporality, spatiality, intersubjectivity, and embodiment), all elements of lived experience are acknowledged and explored, not just those which feature most prominently within transcript data (Rich et al., 2013). Therefore, in utilising the four existentials of the lifeworld in the analysis of J’s account, the meaningful interpretation of the lived experience of VKC victimisation and its consequences can be better understood. Such an understanding may contribute to knowledge that informs the treatment of victims, the understanding of the consequences of VKC, and the prevention of VKC reoccurrence.

## 3. Results and Discussion

**Figure 1:**
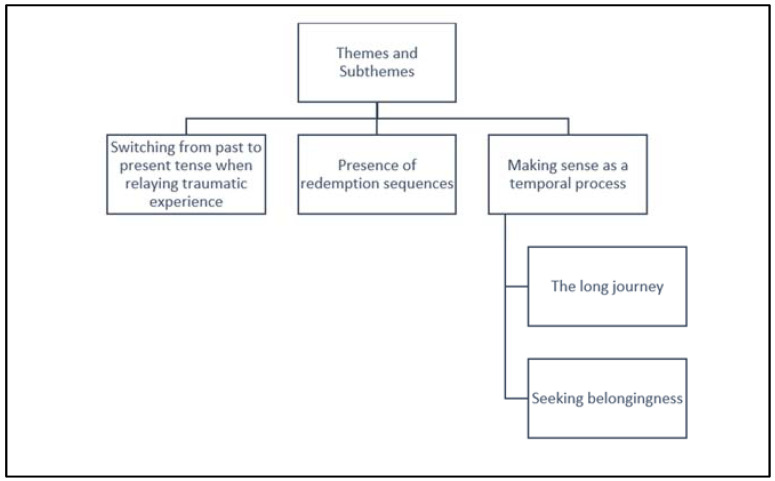
Diagram depicting identified themes and subthemes within J’s interview transcript.

In following the procedural guidance as outlined by Smith et al. (2009) and in the application of the aspects of the lifeworld—temporality, spatiality, intersubjectivity, and embodiment—three themes and two subthemes were identified (see Figure 1).

In his description of the events immediately prior to and at the commencement of the attack, J explained that there had been no interaction between himself and the offender for several months before the incident. He explained that on the evening of the incident, the offender surprised J by visiting him at his home, unexpectedly.

J further explained that the two spent some time watching television and chatting. The events immediately prior to and at the beginning of the attack are described in J’s own words:

*“He just says to me. ‘Ohh, I’d like to give you a massage.’*

*I said ‘OK’, so we’re on my sofa, so I lie on my stomach face down and he gets right on top of me. So, he’s sort of straddled me. I’ve taken my top off. And he starts to massage my shoulders. And at that moment it’s just a little bit… not rough but not massaging, he said. ‘How was that?’ I said ‘Oh, just further up here. Do that bit there’. And he says, ‘How’s this?’ And I [sic]…, ‘That’s better’. Then he says. ‘And how’s this?’ And then when he says, ‘How’s this?’ It felt like he’d punched me.*

*I’m, I’m on, face down because I, I can feel myself lifting up almost even though I got his weight on me. It’s like punched me in the side there. At some point my body slightly moves around and all you can see is his hand there. And… all of a [sic]… I just start to see blood forming around his hand and what I’ve realised at some point, he’s holding a blade of a [sic]…, and he’s stabbed me really high up. Sort of like, chest level on my side, almost round like that where I was, I lay flat down.”*

J goes on to detail a prolonged attack of a 20–25 min duration. In addition to being stabbed, he was bitten, strangled, suffocated, gagged, and bludgeoned with a hammer.

### 3.1. Switching from Past to Present Tense When Relaying Traumatic Experience

An interesting feature of J’s narrative is a distinct switch from past tense to present tense as he relives the more traumatic elements of his experience. When J is describing the non-traumatic, contextual elements of his experience he does so in the past tense:

*“I was the ground floor flat and there was a knock at my window, and I just had some blinds, so I pulled the blinds to one side, and I saw this face and it took me a minute to think who it was. Then I realised who it was.”*

However, as J begins to detail the moments preceding the attack, where he begins to interact with his attacker, he switches from past tense to present tense:

*“And. He. So, then I have to go to the front door. The, the main, it’s a shared, shared entrance and he’s there and he says, something like to paraphrase, Erm… I was. I thought… I was in, you know, I was in the area. I just thought I’d pop in and say hi.”*

In relation to the attack he experienced, J uses entirely present tense descriptions. The use of present tense language within his descriptions, as he relives his experience, gives insight into J’s experience and sense-making in terms of spatiality and temporality. In using present tense language, it is clear to the researchers that J has situated himself closer to the memory of the attack in both space and time and is experiencing these dimensions exactly as his memory indicates they occurred. The experience is imminent.

Dramatic shifts from past to present tense have previously been found to occur in trauma narrations at the point in which a threat to life becomes apparent, suggesting a shift from narrative-based memory to image-based (Pillemer, 2009). Specific imagistic memories, in relation to traumatic experience, are linked to higher levels of wellbeing via the narrator’s flexibility in linking memory specificity and coherence to conceptual structures of the self through meaning making (Singer et al., 2012; Vanderveren et al., 2017). In contrast, difficulties in the recall of specific memories is linked to Post-Traumatic Stress Disorder (PTSD) and depression (Vanderveren et al., 2017). J is reliving the event in his memory and in doing so he provides an authentic account of his sense-making during the attack ‘in the moment’. In addition, his present tense recall demonstrates a level of memory specificity that suggests a potential for the presence of positive trauma outcomes, current wellbeing, and the effective functioning of the self.

### 3.2. Presence of Redemption Sequences

The narrative concept of redemption is useful in making sense of J’s account of his experience of VKC. Redemption describes a situation whereby circumstances evolve over time from negative to positive (McAdams, 2006). Smith et al. (2009) assert that storytelling holds personal significance, and that it is a fundamental aspect of what it means to have an identity. According to McAdams (2019), narrative identity arises from telling stories, and storytelling is grounded in social interaction. As McAdams and Bowman (2001) explain, redemption sequences are a narrative strategy employed by individuals to facilitate sense-making during life transitions; thus, the sequences embedded within the stories of individuals are indicative of the ways in which individuals make sense of personal experience. Redemptive sequences were first identified through J’s many references to being lucky throughout his life, an example of which is detailed below:

*“So, I’m, I’m lucky in some points if I’m born one generation earlier, then you know I’d never have the opportunity to sort of be authentic.”*

In referring to himself as being lucky, J reflects on his experiences from a position of positivity as opposed to focusing on more negative circumstances in which he may describe himself as unlucky. Redemption sequences are evident throughout J’s narrative account. In his description of the attack, redemption sequences are notable in J’s descriptions of being forensically aware and, in accepting the gravity of his situation, making attempts to ensure there is evidence to secure justice and achieve a positive outcome in this way. Through the concept of embodiment, J explains:

*“There’s other little things I remember distinctly, during the attack I took loads of his blood in my mouth.*

*And I remem… it’s so weird, I remember distinctly… I turned to the wall and spat it on the wall. Because I thought ‘I’m gonna die’, but at least his DNA will be. Will be there.”*

Later, in describing the incident itself, he concludes the narrative of the attack with the positive outcome of the offender being apprehended almost immediately and referencing his luck at being in close proximity to a hospital:

*“My attacker flees out the flat with the hammer swings at the neighbour, swings and runs out, but the police pick him up on the next road away. You know he’s; he’s made it around one corner, and they’ve picked him up, someone running away.”*

*“…it was dialled in as maybe a violent thing, it was, there was these, erm, first responders. Which is the. [Redacted] police and they come in with, with guns, you see, because they said that there might be an attack going on anyway… the attackers gone. He’s been picked up then quite quickly then there’s the ambulance people are there. And then. They sort of attend to me. What they can there, mainly stop the bleeding. And then some point soon after… Luckily, I wasn’t too far from the hospital…. I, I’m taken to hospital.”*

Narrative accounts that tend to follow redemptive scripts and describe difficult experiences in a transformative way are related to higher levels of happiness and wellbeing in the narrator (Bauer et al., 2008). Additionally, redemptive processing has been found to be a predictor of psychological adjustment (McAdams et al., 1997) and wellbeing (McAdams et al., 2001). The redemption narrative is clear when J discusses his current circumstances which he describes positively, thus indicating an overall positive outcome despite his negative experiences:

*“Now I’m back on my feet. Obviously, I’m working again. I’ve got my own place again. Erm, I’m very proud of myself, for get… for managing that. I really am. But I think I’m more. But then I realised I am lucky, whether it’s just people around me or some… integral spirit in me that’s got there, but some people are not that lucky that some people are victims of crime, and they never get back on their feet.”*

Despite experiencing extreme trauma and the physical and mental health difficulties he experienced as a result, J is now in a positive place in his life. With consideration of the SIMIC, J’s experience evidences a recognition of identity loss following his victimisation when he describes the loss of employment, home, and relationships by some victims. Additionally, J states that he is back on his feet, suggesting that he experienced significant losses himself which he has since overcome. Indeed, he later describes his loss of employment due to his physical health following the attack, and his relocation back to his hometown, demonstrated below through spatiality, as J describes physically leaving his previous workplace, home, and area of residence:

*“I had to then sort of, leave my job. Which meant leaving my flat. Which meant leaving [Redacted]…and I’m glad now I’m back here, at the time, it didn’t feel that way. I felt like. I felt a direct correlation between. Being a…. someone trying to kill me and being homeless.”*

Such significant changes indicate that J experienced the loss of multiple social identities in the days and months following the attack, including those related to his work life and those established in the area he lived. However, J recognises the important role of others when he states ‘whether it’s just people around me’ while referring to his ability to get back on his feet. It is unclear whether the people he refers to are representative of old or new social identifications; however, what is clear through J’s passing reference is that he perceives his relationships with those around him as a positive influence on his recovery.

Identification with social groups has been shown to positively impact stressful experience because it allows access to social support (S. A. Haslam et al., 2005). Furthermore, research suggests that individuals are more likely to provide and accept social support when group identification is present (Levine et al., 2005). Social support efficacy is increased when it is provided by ingroup members (S. A. Haslam et al., 2004) and research indicates that lower neuroendocrine stress reactions occur when social support is provided by a committee with whom participants have established shared social identity (Frisch et al., 2014). Thus, evidence suggests that social support acts as a buffer to stress, facilitating coping. In alluding to people around him, J is indirectly referencing his ability to access social support, thus facilitating his ability to cope following traumatic experience and move on positively.

Another interesting feature of the above extract is that it offers perspective into the extent to which J internalises victim social identity. J states that he ‘felt a direct correlation’ between ‘Being a…. someone trying to kill me’ and in doing so he seems to demonstrate reluctance to refer to himself as a victim. Evidence shows that men’s recovery from traumatic experience is facilitated by the transformation of the self-view from that of a victim to that of a survivor (Ralston, 2020). While this research highlights such processes in the context of sexual victimisation, the same processes may explain J’s hesitation towards using the word victim within the extract. It is logical to suggest that J does not currently internalise victimhood via self-categorisation due to his reluctance to refer to himself as a victim. Research suggests that individuals who self-categorise as elderly are more likely to believe that they experience symptoms of hearing loss (St Claire & He, 2009), suggesting the opposite is true for those who do not self-categorise as elderly. J’s demonstration of a lack of self-categorisation as a victim may suggest that he does not experience the full extent of the associated negative consequences of victimisation at this point in time. This may therefore indicate the presence of recovery, reduced symptoms of PTS, and potential PTG.

Within the transcript, J references his contact with services and processes targeted towards victims, namely victim support services and engagement in the parole processes:

*“And even though I made contact with Victim Support at the time, they didn’t do like… erm… I can go, I could go and see someone or they, they would recommend a therapist”*

*“I’m given the, I’m able to update my impact statement or in the unlikely event he got released I could do something called a non-molestation order which meant I could restrict where he could travel.”*

In line with research which suggests asthma sufferers are more likely to comply with medication if they internalise their diagnosis through social identity (Adams et al., 1997), J’s willingness to engage with victim support and parole processes may suggest that he did at one time internalise victim identity. This appears to no longer be the case, seemingly substantiating the findings of Ralston (2020) that recovery in men is facilitated through the transformation of the view of the self from that of a victim to that of a survivor. Such an interpretation, consistent with the SIMIC, further evidences the presented argument that recovery from traumatic experience is a process of social identity transformation.

Arguably, one significant turning point within J’s narrative account is the decision he took to support the potential deportation of his attacker. A difficult decision which caused conflict for J, described below, through embodiment, where he discusses his thoughts in terms of what his decision did to his head, and through expressing his decision as his voice. In addition, according to Damasio’s Somatic Marker Hypothesis (1996), cognition, or thought, is an embodied process which occurs physiologically in the brain and which invokes feelings and emotions leading to reasoning and decision making. J’s description of his thoughts and related emotions that drove his decision making with regards the deportation of the offender are thus communicated through the concept of embodiment:

*“Erm, they, they did ask me what I thought, whether I would support them going for a deportation. Erm and it’s such a weird thing because I thought, gosh, after those years I could say ‘Yes, deport him’ knowing he’d be released, now that, that did two things to my head. One thing was. My thought of… him… erm, being free again and what, he could hurt somebody again. So, by me by me supporting it. Not that they would have… [sic] I was the deal maker, but I imagine my decision… My voice mattered. Or was the deciding factor… [sic] thought ‘Can I live with the idea that he’s gonna be released and hurt somebody again?’*

*And then I thought, gosh, and also. It’s an act of kindness. I could actually do something that could give him his freedom. How, sort of, perverse is that?”*

J continues his discussion around deportation by referencing the unlikelihood of his attacker being released due to his behaviour while incarcerated:

*“And so, I, and he was so far off ever been released, you know, he was showing violent behaviour in prison to prisoners, to prison guards. Wouldn’t engage in prison programs for reform. Would not erm, take full responsibility for the attacks. Still, after years, so he was gonna rot in there. And this lifeline was thrown him to be deported and I said. ‘Yeah, deport him.’”*

This extract is interesting from a social identity perspective. J’s knowledge that the offender was engaging in problematic behaviour, enables him to access the offender’s inner world and is thus intersubjective in nature (Harrison & Tronick, 2022). Having access to this information may have enabled J to employ a strategy of dehumanisation and depersonalisation of the offender which enabled him to firmly position the offender in the category of ‘other’. The view that the offender is displaying problematic behaviour offers J the possibility of depersonalising his attacker as the prototypical perpetrator of violence. Research suggests that when perpetrator status is afforded to the outgroup, dehumanisation is possible due to the perception of low morality (Formanowicz et al., 2023). Such a strategy may be conceptualised as adaptive depersonalisation and dehumanisation, freeing J from the intersubjective ties to the offender, and thus enabling him to enact the ultimate process of othering, by supporting deportation and the physical removal of the offender from the country.

Ultimately, despite his inner conflict, J’s decision to support deportation allowed him to move on from the repeated re-traumatisation of engaging with criminal justice procedures. J describes this moving on with reference to the intersubjectivity of no longer engaging with criminal justice professionals and court proceedings, ending that chapter of his journey, and freeing him completely from the intersubjective connection to the offender:

*“And that does feel more final now because there’s no more parole hearings. There’s no more things, no more victim impact statements for me to write. So, it did feel like. Something finishing it did feel like a back of a book closing.”*

In describing this event as ‘something finishing’ and ‘like the back of a book closing’, J demonstrates that he understood this situation as transformative and a turning point from which he was able to move on more positively. Research suggests that the ability to narrate causal turning points within narrative accounts is associated with current and long-term wellbeing (Mitchell et al., 2020).

The presence of redemption sequences throughout J’s account, and the overarching redemption narrative across his biographical account, indicates J’s ability to be adaptable and to maintain hope and confidence when facing difficult situations in his life (McAdams & McLean, 2013). This is evidenced below, where J discusses how his experience has changed his view of the world in positive terms:

*“It has, it has, changed and I do I think I’m more compassionate now, which is a strange thing to say, I suppose”.*

Additionally, J’s frequent use of redemption sequences (Bauer et al., 2008) along with his use of causal turning points (Mitchell et al., 2020) demonstrates his tendency towards positive outcomes, enabling him to reframe his experience as one which is transformative, giving him renewed purpose (Cook & Walklate, 2019). As demonstrated below, where J describes his progress in terms of increased empathy and connection to others, highlighting the intersubjective nature of his experience:

*“I’m not really paying much attention to the news recently, so I’m not tuned into that one, but occasionally it just, one of them clicks like and it just and it lingers in my head. It’s like a bit of an intrusive thought. Erm, but so like I was saying, I think I’m a bit. I have a bit more empathy. I didn’t for a while. I think after my attack went the opposite, where I was a bit self… absorbed. And not the best friend. I don’t think for a long time. But then I think since I feel like I’ve come out the other side slightly, is that I feel more empathetic now. Erm, so, in other words I got back at it, took me a lot to get back on my feet.”*

This indicates the presence of positive psychological wellbeing, psychological adjustment, and a degree of happiness on J’s part at this point in time. While J attributes this to being ‘lucky’, he makes passing reference to the people around him, and the types of social identity loss that he experienced, suggesting that he recognises the importance of social belonging and support with regard to his recovery. J’s experiences of PTS and his tendency to narrate experience and specific memories into wider frameworks and redemptive sequences appears to have facilitated PTG, through increased connection to others and social identity gain. Therefore, the effectiveness of enabling victims of VKC to frame their own experiences within redemptive sequences may be a beneficial tool within their treatment. In addition, the experience of social identity loss may be reduced within treatment by including strategies that enable the development of new social identities via support groups (Muldoon et al., 2019a).

### 3.3. Making Sense as a Temporal Process

The recovery from traumatic experience can be understood in the context of searching for new meaning following adverse experience (Joseph, 2013). The inability to make sense of traumatic experience is suggested to impede the healing process, if not make healing impossible. Academic literature suggests that emotional processing and sense-making of traumatic experiences can reduce adverse psychological symptoms and can positively impact wellbeing (Greenhoot et al., 2013). Much like cognition, and relative to the position of IPA, which argues that emotion is embodied (Smith et al., 2009), Damasio et al. (1996) argue that emotion is an embodied phenomenon, influencing reasoning and thus facilitating meaning-making. Additionally, meaning-making, in which individuals can make sense of their experiences and identify positive outcomes, has been shown to be positively correlated with post-traumatic growth (Larsen & Berenbaum, 2015). For J, his attacker’s continued denial of responsibility has likely inhibited his ability to make sense of why he was attacked and thus his sense-making and post-traumatic growth is an ongoing process. J’s emotional discomfort regarding the offender’s denial and not-guilty plea is demonstrated below. Within the extract, J makes specific reference to the passing of time, highlighting the temporality of this situation:

*“Erm, so yeah, it would’ve been nice if he said guilt…, he pleaded guilty, but it so that that was quite a long. That was a very difficult process because it was erm, I was in the stand two days, one day with the Crown barrister, sort of like on my side I suppose and then this defence barrister.”*

J’s continued efforts to make sense of his experience is demonstrated throughout his account, and when reflecting upon his experience he states:

*“I won’t feel any anxiety about it, I suppose. It’s just a thing I just reflect upon really. Like my cenotaph in my head… Really. Where, I lay a wreath. Erm and pause and think really. Yeah.”*

In describing ‘my cenotaph’, J makes a very symbolic statement which, by referencing a war memorial, suggests that he makes sense of his experience as one which has been a kind of personal war. The experience is a part of J and making sense of the experience is an ongoing process for J which will continue throughout his life. In noting a lack of anxiety when he reflects on the incident, J indicates that this type of reflection is not associated with negative emotional responses and distress, indicating that post-traumatic growth may be present and ongoing (Larsen & Berenbaum, 2015). This type of reflective rumination is said to be characteristic of a search for meaning and a reframing of experience into the life narrative (Joseph, 2013).

Event centrality is a concept that refers to the extent to which a person incorporates a traumatic event and perceives it to be central to their identity (Boals & Murrell, 2016). J’s discussions of his experience of time following the incident are indicative of his initial tendency to conceptualise the event as central to his being, as demonstrated through temporality in the extracts below, where J directly references periods of time since the attack occurred, and his revisiting of the incident at 8pm, the time the events unfolded:

*“It’s strange the first year after my attack the…. This is very interesting; I’ve found is that. I was probably not conscious maybe three or four days after and when I was conscious and every night… Let’s say 5 days post attack you got to 8:00 PM. It would overwhelm me, this time 5 days ago I was being attacked. Then it got to a week anniversary. This time last week I was being attacked and then it got to the first year, you know, I think. Oh my God. 8:00 PM, he’s knocking on my window now”.*

The above extract demonstrates J’s tendency to reexperience the incident initially; however, J goes on to state that this has lessened over time, and he no longer experiences any anxiety when he does think back to the exact time when the event occurred:

*“Now, when I reflect back on it, that’s a massive part of it, but I often just feel a lot more of a… Collective emotion over the last (redacted) years I suppose, and the journey that I’ve been on. And, and I just, I don’t feel incredibly sad, but I just sort of. You know, think about it. Think about things, people. I’ll find a quiet time.”*

*“I still think at 8:00 PM, by the way, I still, it’ll still happen to me. I’ll be thinking tomorrow, 8:00 PM tomorrow night thinking ‘ohh’. But it won’t be. I won’t feel any anxiety about it, I suppose. It’s just a thing I just reflect upon really.”*

While J has certainly incorporated his experience of the attack into his life narrative and acknowledges difficulties he has experienced since the attack, he does not appear to view it as the central aspect of his identity that it once was. However, it does influence the way in which J interacts with the world and others, as described below, giving insight into the spatiality of his experience, through his perceptions of the places he may encounter risk:

*“Erm… so my brains never been the same since, I am absolutely fine, by the way, but there’s definitely it’s not something I feel like I’ve. In the past, got over, end of, it’s definitely fed into some of my things I do now, even now, erm, for no reason sometimes I can just get a little bit…. Erm… see risk in places that are not there. Like walking past, you know, some scaffolders. I think ‘Oh God, someone’s gonna drop a hammer on my head’”*

Although J states he does not feel anxious when reflecting on his experience, he does describe embodied distress as he begins to describe and relive details of the event itself when he states, ‘so this, I suppose, [is] a bit more difficult for me’ and ‘Just my breathing goes a bit funny sometimes’. This type of reflection positions J closer to the incident in time and space, to a point where the event was central to J’s being, the point at which the event was being experienced and is demonstrated by a switch from past to present tense narration. While it may seem paradoxical, the literature indicates that the periodical cognitive revisiting of traumatic experience and reflection upon ‘what is lost’ may be necessary in the maintenance of personal growth following traumatic experience (Henson et al., 2021). Reflection, although potentially unpleasant, emphasises that which has been gained over time (Henson et al., 2021). Furthermore, repeated reflection upon adverse and traumatic experience can be understood in the context of attempting to make sense of the experience, accepting the new reality following the event and incorporating these aspects into the life story (Joseph, 2013). With this in mind, the SIMIC may offer some understanding as to the role of social identity and personal growth following traumatic experience. Reflection upon social identity loss may highlight the positive aspects of social identity gain that have occurred since the traumatic experience and repeated reflection may enable victims to accept their new social realities more readily. Therefore, the consideration of the manner in which victims of VKC reflect upon their experience may be useful in the construction of treatment strategies and thus warrants further investigation. In addition, victims should be deterred from avoiding unpleasant memories, but may instead benefit from strategies that enable reflection in way that promotes the acceptance of new social identities and PTG.

#### 3.3.1. Subtheme—The Long Journey

The ‘long journey’ was identified as a subtheme and demonstrates that J makes sense of his experience as one which has shaped his life over a long period of time and as one in which he continues to seek meaning through the temporality of his experience. To demonstrate this ongoing ‘long journey’, J makes references to his experiences of time throughout his account. These are most evident when he discusses his physical recovery and criminal justice procedures. This appears to enable J to emphasise that both of these aspects of his account, to him, seemed to occur over prolonged periods of time. This also enables J to document these long processes in terms of his progress across time which resulted in positive outcomes. As such, J’s experience of time is framed within redemptive sequences and causal turning points are included within these sequences as specific points in time; thus, J indicates the temporality of his experience.

*“I was in the stand two days, one day with the Crown barrister, sort of like on my side I suppose and then this defense barrister. And then he took the stand and eventual long story cut short. After three weeks, the jury goes out and he was found guilty of attempted murder. And then…That was the [date redacted]. And it’s come back for sentencing. And we came back nine times for sentencing, and it was the following [date redacted] he finally got his sentence of [sentence redacted] years.”*

Here, each reference to time appears to emphasise the ‘long journey’ of J’s experience; however, it also refers to a particular hurdle that J must overcome to progress on his journey. In achieving each milestone, J moves closer in time towards the positive outcome of a guilty verdict and a prison sentence for the offender. J’s ‘long journey’ is also demonstrated in his projection of his experience into his perceived future, particularly with regard to his reluctance to pursue intimate relationships giving insight into intersubjectivity:

*“Also, the other one is intimacy, of course, is that I’ve… that I did have a I did end up dating someone for a very short time, that didn’t work. And, about six years ago, I decided right. That’s it. I’m not gonna let anybody… I’m not gonna date anyone, no one’s gonna touch me.”*

In the same way that narrative identity states that experience incorporates the past and informs the expected future (McAdams & McLean, 2013), the attack that J experienced is incorporated into his experiences and sense-making and informs his expectations of his own future, in which he anticipates continued reluctance to engage in intimate relationships. J’s descriptions of intersubjectivity represent a highly significant example of social identity loss which J has not yet been able to renew and has resulted in social identity discontinuity. This indicates an area in J’s life in which he continues to experience PTS (Muldoon et al., 2019a) which manifests as the avoidance of intimacy.

While J can look to the future in a positive way across certain domains—for example, when discussing his career—J’s expectations regarding future intimate relationships are viewed in a more negative light. Intimate relationships represent a part of J’s long journey yet to be resolved, which may continue for some time. Analysis of J’s long journey offers insight into the ways in which victims may utilise causal turning points within their narratives or project particular difficulties into their futures and may be indicative of the areas in which PTS is present and where PTG is possible.

#### 3.3.2. Subtheme—Seeking Belongingness

While J’s difficulties around intimate relations are evident within the subtheme ‘The Long Journey’, they are also highly significant to the subtheme ‘Seeking Belongingness’. One particularly insightful quote demonstrates J’s understanding of the attack as an extreme abuse of trust on the part of his attacker. This abuse of trust is assimilated and projected by J into his future narrative, where he makes sense of future intimacy as a potential threat. By projecting this uncertainty around intimate relationships going forwards, demonstrated in the extract below, J indicates this as an area he continues to try to make sense of and offers insight to the intersubjectivity of his experience, though his projection of expected outcomes related to intimate relationships:

*“My life plan right now is not to have… Anybody… Not to be intimate again, or let, put myself in… it… because it was very intimate what happened to me”.*

This pattern of distancing from others is one previously adopted by J at the time of him making sense of his sexuality indicating consistency in communion, or connection to others, across J’s lifespan narrative. This suggests that such distancing and avoidance may be a pattern of behaviour that J has adopted previously at a time when he has felt that social relationships may threaten his safety and wellbeing, potentially resulting in social identity loss. According to the SIMIC, social identity loss impacts domains such as health and wellbeing because it compromises access to social support. These notions are evidenced through academic research which highlights the importance of social identity loss and gain within contexts such as retirement (C. Haslam et al., 2019), during transition from adolescent to mental health services (McNamara et al., 2017), and when understanding health and wellbeing outcomes following mental and physical health diagnoses (Barker et al., 2014; Conneely et al., 2020; C. Haslam et al., 2008; Walsh et al., 2020). Where social identity loss is experienced, the negative consequences on health and wellbeing are accentuated (C. Haslam et al., 2008) through a lack of access to social identity resources. Within J’s account, his avoidance of intimate relationships highlights a significant social identity loss and is an area in which he continues to experience PTS. In seeking belongingness, J attempts to address his social identity loss through social identity gain with others, thus enabling access to social support to facilitate recovery, alleviate PTS and potentially working towards PTG.

Avoidance-orientated approaches to coping following a traumatic experience, while somewhat problematic, are fundamental to post-traumatic stress which, in turn, is necessary for PTG to occur (Joseph, 2013). The presence of PTS in the domain of intimate relationships is not surprising when it is considered from a social identity approach. According to Muldoon et al. (2019a), PTS is heightened when traumatic experience threatens valued social identity. J’s trauma occurred at a time when he was able to be open about his sexuality and, during a very intimate moment, a context in which his sexuality likely became a salient social identity. Following the attack, J’s experience of discontinuity and loss around intimacy and sexuality continues to be an area in which he experiences tension and uncertainty. The attack he endured represents a threat to that social identity, and a significant social identity discontinuity between his old-self and his new reality. This impacts the sense of belongingness that J has sought since the attack, and represents his need to access social identity resources in the form of support, solidarity, and perceived control (C. Haslam et al., 2021). J therefore maintains control through his continued avoidance of intimate relationships.

While J makes sense of others as a threat in an intimate capacity and distances himself as result, his intersubjective experience is further indicated as he explores his desire to seek affinity, connection, and solidarity, albeit with other victims of violent crime. In other words, J seeks out social belonging with other victims, in an effort to address the social identity loss and access to social identity resources experienced through the avoidance of intimate relationships:

*“…one thing I really was, I sought like, was, was, trying, trying, trying, to find somebody who’s been through something similar.”*

In terms of PTG, this indicates a time whereupon J explored his ability to increase his connection to others and demonstrates increased compassion for other victims (Kinsella et al., 2015). J’s desire to connect with other victims represents his attempts at social identity gain. J attempts to forge new, meaningful social identities, which are suggested to enhance resilience and increase the likelihood of PTG (Muldoon et al., 2019a). Research indicates that the formation of new valued social identities is vital to positive recovery outcomes (Jones et al., 2012; Muldoon et al., 2019b). Through connecting with others, J makes efforts to make sense of his own experiences through shared understanding; he states “I wanted maybe to, to talk to somebody and have somebody and just look at them and say “ohh you know””. J explains this was something he sought out initially but was unable to find other victims with whom he could connect. Social belongingness is a mediator of PTG (Levi-Belz, 2019) and, in seeking social belonginess, J demonstrates his ongoing efforts to make sense of his experiences through validation, shared experience, and a shared social identity with others. In other words, following multiple significant social identity losses and discontinuity, J actively sought out new shared social identities which diminished his stress and facilitated PTG through connection with others.

Group identification is suggested to enable individuals to regain control of individual narrative identity, something that can be perceived as being lost following trauma (C. Haslam et al., 2018). Further, group membership that arises in the context of the experienced trauma provides social support and a sense of purpose, enabling positive development (C. Haslam et al., 2018). Applying J’s experiences of seeking belongingness to the treatment of victims of VKC highlights the potential need for victims to establish social identity with individuals with similar experiences. Victim support groups may be impactful in this endeavour (Muldoon et al., 2019a); however, potential rivalries between the victims of VKC may impede the effectiveness of support groups. It is therefore necessary to consider ways in which victims can establish a sense of belonging in manner which prevents exposure to rivals, revictimisation, and/or future offending.

### 3.4. Strengths and Limitations, Implications, and Directions for Future Research

The current study has given insight into the psychological mechanisms that may facilitate PTG following an incident of VKC. In making sense of J’s sense-making of his experiences, the researchers have attempted to capture J’s reframing of his experience into positive outcomes. The intention was to mirror this reframing in the presented analysis and conceptualising this in terms of PTG enabled the researchers to demonstrate J’s tendency towards the positive and, in doing so, demonstrate his sense-making as being a temporal process. A particular strength of the study was the transferability of the research findings to victims of traumatic experience including VKC. The understanding that PTG and recovery from traumatic experience is an ongoing process is important when considering tertiary prevention strategies that may help reduce VKC. J’s continual recovery process demonstrates the need for practitioners to continually work with victims. Within the context of VKC, this is particularly important given the extent to which victim–offender overlaps occur (Bailey et al., 2020) and evidence which suggests previous victimisation to be a risk factor for future offending (Haylock et al., 2020). Treatment of victims which promotes PTG may be effective in preventing future knife-related offending, and the incorporation of elements such as redemptive narrative framing, social identity continuity and gain, and a shared social identity that promotes positive outcomes may be effective strategies. Such an approach is relevant to tertiary prevention strategies of a public health approach to tackling knife crime, which focuses on understanding the consequences of VKC after its emergence to inform strategies to prevent its reoccurrence (Neville et al., 2015). That said, such assumptions, based upon the analysis of a single case, should not be seen as definitive and instead the presented analysis should be seen as exploratory, and one which gives perspectives from which to devise future research.

A limitation of having retrieved such rich data derived from J’s account meant that there was too much data for a single study. J’s experiences of the criminal justice system are an example of experiential data that were beyond the scope of the current analysis. Analysis of these aspects of J’s experience would undoubtedly allow deeper understanding of specific difficulties experienced by victims of traumatic crimes who encounter criminal justice procedures that may contribute to retraumatisation. Therefore, a recommended direction for future research is the experiential study of encountering the criminal justice system following traumatic victimisation. Additional directions for future research may include research that explores the conclusions presented in the current analysis, that recovery from VKC may be a process of social identity transformation. Additionally, investigation of the effectiveness of the implementation of redemption narrative-framing and the promotion of shared social identity within the treatment of victims of VKC. Such strategies seem to have facilitated J’s PTG following his experience and may therefore have a positive impact on other victims.

The current research may also raise further questions as to the transferability of the findings to victims of other forms of trauma and whether they too can be conceptualised as processes of social identity change, and may therefore provide directions for further research. In addition, the experience of trauma is not only confined to the victim experience. Research indicates that victims, witnesses, and offenders are vulnerable to the psychological impact of traumatic experience (O’Donnell et al., 2011; Soh et al., 2023). Therefore, the experiential study of the offender’s and witness’s lived experience of traumatic violence maybe a fruitful avenue of investigation which gives further insights into the psychological impact of trauma and the contextual similarities and differences in their experiences.

## 4. Conclusions

The current study provided insight into VKC utilising a single case study. J’s account of his experience of an extremely violent attack generated transferable knowledge related to the ongoing sense-making of the victims of violent crimes. The research question ‘What is the lived experience of VKC?’ is answered via J’s account. The lived experience of VKC goes beyond making sense during the incident itself and continues across time. J’s experience highlights the significance of redemptive processing in recovery and the importance of acknowledging traumatic experience as transformative to social identity. Due to the ongoing nature of recovery from VKC, to focus only on the violent event is to neglect the full experience of traumatic violent victimisation. Indeed, much of J’s sense-making is seen to take place in his experiences since the event. J’s experience highlights the ongoing impact of traumatic experience as he continues to make sense of the incident and its psychological effects. The mechanisms J utilises to make sense of the incident include reframing of experience into redemptive narratives, and acceptance and the formation of new shared social identities. These mechanisms have seemingly enabled J to achieve positive outcomes following the traumatic experience; therefore, they may provide a framework from which victims of VKC can be researched in future which, in turn, may inform how they are treated as part of tertiary prevention strategies within a public health approach.

## Data Availability

Transcript data contains identifiable information and is thus not available to preserve the anonymity of participant ‘J’.
